# Intraspecific Variation in Elemental Composition of the Least Killifish Tracks Spatial Variation in Periphyton Composition

**DOI:** 10.1002/ece3.72326

**Published:** 2025-10-13

**Authors:** Benjamin D. Pluer, Pamela S. D. MacRae, Joseph Travis

**Affiliations:** ^1^ Department of Biological Science Florida State University Tallahassee Florida USA; ^2^ College of Arts and Sciences University of Maine at Augusta Augusta Maine USA

**Keywords:** ecology, freshwater ecology, freshwater ecosystems, freshwater fish, interspecific variation, periphyton, poeciliid, stoichiometry

## Abstract

Many animals, especially freshwater fish, display significant population variation in elemental composition. How often that variation reflects patterns of interspecific variation remains under‐explored. We examined the elemental composition and trophic niche of *Heterandria formosa* , an omnivore whose trophic position varies among populations. We studied eight populations, four from freshwater springs and four from shallow lakes. We analyzed water chemistry along with elemental composition and stable isotopes of carbon and nitrogen in periphyton and fish to determine the extent of population variation in elemental composition and any associations it might have with variation in the composition of the basal resources or the trophic level of the fish. Water chemistry varied along a gradient from lakes with lower values of pH, higher concentrations of chlorophyll‐a, and lower nitrate concentrations to springs with higher values of pH, lower chlorophyll‐a concentrations, and higher nitrate concentrations. The population variation in elemental composition of the fish tracked the variation observed in the periphyton samples. There was a narrow range of C:N ratios among populations, compared with studies of other species, but differences among them were statistically significant. There was little variation in C:P and N:P ratios as well. Mantel analyses revealed that differences between pairs of populations in elemental compositions of fish were not correlated with differences in the water chemistry. The differences between pairs of populations in the percentages of carbon and nitrogen in fish were strongly correlated with the pairwise differences in those variables in the periphyton, indicating that the elemental composition of the fish tracks that of its basal resource. The average values of *δ* ^13^C in the fish populations were highly correlated with the average values of *δ* ^13^C in the periphyton samples at those locations. Fish populations in springs, which experience less predation pressure and display higher population densities, occupied lower trophic positions than populations in lakes, which experience more predation pressure and exhibit lower population densities. The association between the elemental composition of fish and periphyton could be driven by either bottom‐up or top‐down effects. The constancy of elemental composition despite variation in trophic position contrasts with well‐described patterns of interspecific variation. This might reflect the limited range of variation in trophic position among populations compared to the ranges observed in interspecific analyses.

## Graphical abstract

We examined organismal stoichiometry and trophic niche of *Heterandria formosa* , a periphyton grazer, in eight populations, four each from freshwater springs and shallow lakes. Water chemistry varied along a gradient from lakes to springs. Population variation in organismal stoichiometry of fish tracked the variation observed in periphyton samples. Associations between the organismal stoichiometry of fish and periphyton could be driven by either bottom‐up or top‐down effects.

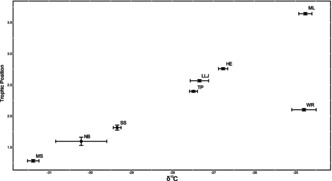

## 1 Introduction

Ecological stoichiometry, the mass‐balance relationships in elemental composition among species in an ecosystem, is fundamental to understanding a wide variety of ecological phenomena, from foraging patterns to nutrient cycling (Sterner and Elser 2002). Variation in organismal stoichiometry, be it interspecific or intraspecific, can drive substantial variation in community and ecosystem dynamics (El‐Sabaawi, Marshall, et al. 2015; Leal, Seehausen, and Matthews, et al. 2017).

Interspecific variation in organismal stoichiometry can be particularly important in aquatic systems, in which the elemental composition of primary and secondary consumers can be quite different from those of their resources (McIntyre and Flecker 2010). This is particularly true when considering fishes, which display considerable interspecific variation in organismal stoichiometry (Hendrixson et al. 2007; Andrieux et al. 2021; May and El‐Sabaawi 2022) and account for a substantial proportion of the nutrient cycling in many stream habitats (Grimm 1988; Vanni et al. 2002). Some of the interspecific variation can be attributed to structural variation; specifically, the proportion of skeleton and bony tissue, which plays a large role in determining phosphorus content (McIntyre et al. 2008; El‐Sabaawi et al. 2016). Conversely, similarities in body structure among related taxa produce the strong phylogenetic signal in phosphorus content and in the ratio of nitrogen to phosphorus (N:P) (Hendrixson et al. 2007).

Many fishes display considerable variation among conspecific populations in elemental traits (El‐Sabaawi, Kohler, et al. 2012; El‐Sabaawi, Zandona, et al. 2012; Ebel et al. 2015; Tuckett et al. 2016; Durston and El‐Sabaawi 2017; Montana and Schalk 2018; Wei et al. 2022; López‐Sepulcre et al. 2024). In some cases, the magnitude of population variation is comparable to measured levels of interspecific variation (El‐Sabaawi et al. 2014, 2015). Some of this variation is induced by environmental differences among populations, like thermal conditions of their habitats (Moffett et al. 2021) or diets (Fagan et al. 2002; Leal, Best, et al. 2017). In many cases, population variation is associated with variation among locations in primary productivity (Downs et al. 2016; Tuckett et al. 2016) or the elemental composition of food resources (Dickman et al. 2008; El‐Sabaawi, Kohler, et al. 2012). There is also evidence that some proportion of observed population variation can have a genetic basis (Durston and El‐Sabaawi 2017; Lemmen et al. 2019).

Several aspects of intraspecific variation in elemental composition are not well understood. For example, there is little consistency in whether elemental traits vary with body size (Sterner and George 2000; McIntyre and Flecker 2010). Some species show strong relationships (El‐Sabaawi, Kohler, et al. 2012; Burress et al. 2013; Boros et al. 2015; Montana and Schalk 2018), while others show none (El‐Sabaawi, Zandona, et al. 2012; Montana and Schalk 2018; Moffett et al. 2021). As another example, how often population variation in elemental composition tracks variation in the basal resources is unknown; while some studies of population variation have examined the basal resources (El‐Sabaawi, Kohler, et al. 2012; El‐Sabaawi, Zandona, et al. 2012), many have not. Finally, it is unclear whether the association between interspecific variation in elemental composition and variation in trophic position (Vanni et al. 2002; McIntyre and Flecker 2010) has a parallel in intraspecific variation. In Trinidadian guppies, populations in which fish fed higher in the trophic web had higher percentages of nitrogen than populations in which fish did more primary consumption (Zandonà et al. 2024). How often this pattern might be found is unknown.

North Florida populations of the Least Killifish, *Heterandria formosa*, offer an excellent opportunity to address these issues. This small (males average about 12 mm standard length [SL], females average about 18 mm SL) inhabitant of the shallow littoral zone is found in nearly every lotic and lentic body of water in the region (Baer 1998; Bagley et al. 2013; MacRae and Travis 2014) and displays a wide range of variation in population density and life history (Leips and Travis 1999; Schrader and Travis 2012; MacRae and Travis 2014).

*Heterandria formosa* is especially suitable because its diet and trophic position vary from one population to another. Analyses of gut contents have indicated *H. formosa* to be a primary consumer at some locations (Hunt 1953; Loftus 2000) but a planktivorous secondary consumer in others (Reimer 1970; Schaefer et al. 1994; Loftus 2000). A stable isotope analysis of trophic position in one north Florida population was consistent with *H. formosa's* being a primary consumer (Aresco et al. 2015). In Trinidadian guppies, populations experiencing higher densities and lower predation pressures fed lower in trophic position (Zandona et al. 2011; Zandonà et al. 2024). North Florida populations of *H. formosa* vary along a similar gradient of high predation/low density to low predation/high density (Schrader and Travis 2012; MacRae and Travis 2014) and so may also vary in trophic position and, perhaps, elemental composition.

The habitats in which *H. formosa* is found in north Florida fall into two main categories. Spring‐fed rivers have alkaline waters (pH values ~7–8) with high chloride content, while the region's lakes have acidic waters (pH values ~4–6) with high concentrations of tannic acids from decaying terrestrial vegetation. There are substantial differences between springs and lakes in the species of diatoms and soft algae found in them (Whitmore 1989; Sweets et al. 1990; Mattson et al. 1995; Stevenson et al. 2004; Fore 2010; Aresco et al. 2015). Periphyton is the base resource in the trophic web of these habitats (Aresco et al. 2015), and the variation in algal composition between habitats suggests there can be substantial variation in the elemental traits of the resource bases used by *H. formosa*.

Here we report a survey of the organismal stoichiometry and trophic niche of *H. formosa* in eight populations, four from each of two contrasting habitat types. First, we describe variation in water chemistry among the eight populations we selected. We also document variation among these eight locations in the elemental composition of periphyton in the littoral zone where *H. formosa* forage. Second, we examine the organismal stoichiometry in those populations. We show that there is less population variation than has been reported in other species but that the population variation that is present tracks variation in the carbon and nitrogen profiles of the periphyton in these locations. Third, we describe the trophic niche of *H. formosa* in those eight populations, establishing that the trophic niches of these fish are consistent with their being primary consumers in sites with lower predation pressure but secondary consumers where predation pressure is higher and density is lower. Fourth, we report the lack of any relationship between elemental composition in the fish and their trophic position.

## 2 Materials and Methods

### 2.1 Study Sites and Collection Methods

We sampled water at 8 locations with populations of *H. formosa* between the years of 2010 and 2013 (Table S1). These locations included springs and open canopy lakes. We selected these eight locations for stoichiometric and stable isotope analyses: four freshwater springs (McBride's Slough, Natural Bridge, Shepherd's Spring, Wacissa River) and four lakes (Harper's Eyelet, Little Lake Jackson, Moore Lake, Trout Pond). We chose these eight locations for two reasons. First, they spanned a wide range of water chemistry parameters (Table 1). Second, the populations of *H. formosa* in these locations displayed a range of population densities and life histories similar to the range seen among populations in the region (Schrader and Travis 2012; MacRae and Travis 2014).

**TABLE 1:** Water chemistry variables at each sampled site in each year sampled. Letters in column 1 denote the site while the numbers denote the year (e.g., “10” = 2010, “11” = 2011, etc.).

Site	Chlorophyll_a	pH	Nitrate	Total_N
HE12	18	6.7	0.012	1.33
HE13	2.49	6.37	0.012	0.49
LLJ12	3.53	6.38	0.012	0.651
LLJ13	6.83	7.14	0.033	0.577
ML10	3.25	5.03	0.012	0.854
ML11	9.02	5.39	0.012	1.25
ML12	4.45	4.98	0.012	1.03
ML13	3.18	5.15	0.012	0.518
MS10	1.51	7.3	0.359	0.609
MS11	1.47	7.48	0.012	0.393
MS12	0.46	7.69	0.072	0.333
MS13	0.25	7.76	0.168	0.337
NB10	0.418	7.07	0.071	0.854
NB11	0.403	7.81	0.279	0.448
NB13	0.25	7.81	0.175	0.342
SS10	1.18	7.46	0.194	0.355
SS11	0.547	7.43	0.06	0.281
SS12	1.66	7.71	0.012	0.305
SS13	7.58	7.84	0.077	0.214
TP10	3.07	5.35	0.012	0.563
TP11	28.7	5.84	0.012	0.536
TP12	12.8	4.7	0.012	0.758
TP13	2.03	5.46	0.012	0.537
WR10	2.1	7.56	0.302	0.527
WR11	0.547	7.89	0.474	0.693
WR12	0.25	7.83	0.119	0.265
WR13	0.25	7.85	0.308	0.45

*Note:* Nitrate and Total N are in units of mg/L; chlorophyll a is in units of μg/L.

We collected fish in 2012 and 2013 via dip netting in the shallow littoral zone, typically at depths less than 0.30 m. We sacrificed fish via rapid chilling in an ice slurry, following approved IACUC protocols. We used samples from 2012 and 2013 for elemental analyses but performed isotope analyses only on fish collected in 2013. We obtained periphyton samples in 2013 by scraping stems and leaves of aquatic plants, larger rocks, and fallen limbs wherever we saw *H. formosa* individuals foraging. We placed all samples on ice for return to the laboratory.

### 2.2 Water Chemistry Methods

We collected water samples by lowering a Nalgene bottle on a long pole into clear water, approximately 1.5 m from the shoreline and away from aquatic vegetation. We filtered the water on site by pouring it through a 10 μm filter and immediately placed the filtered water on ice for transport the same day to Akuritlabs Inc., in Tallahassee (National Environmental Laboratory Certification E81350). From each sample analyzed by Akuritlabs Inc., we obtained data on pH, concentrations of chlorophyll a, total nitrogen, organic nitrogen, nitrite, nitrate, ionized and unionized ammonia, total phosphorus, and orthophosphate. We limited our analyses to pH, chlorophyll *a*, nitrate, and total nitrogen, as only these variables consistently exceeded their respective detection limits (1.0 mg/mL, 0.25 mg/m^3^, 0.012 mg/L, and 0.071 mg/L) across all sampling locations.

### 2.3 Elemental Composition and Isotope Methods

We prepared fish tissue and periphyton samples for elemental and isotope analyses following Aresco et al. (2015). In brief, upon return to the laboratory, we removed and discarded all internal organs, including reproductive tissue, gut, and liver/viscera, where lipids are stored for future use (McManus and Travis 1998). After freeze‐drying the fish carcasses and periphyton, we ground the freeze‐dried material to a fine powder using a WiglBug Model 3110B, rinsing the capsule and grinding bearing with ethanol between samples. For analyses of carbon and nitrogen, we prepared ground material in small tin capsules with, on average, 1 mg fish tissue and 2 mg periphyton. These samples were analyzed using an EA‐IRMS (Elemental Analyzer—Isotope Ratio Mass Spectrometer) system (Elementar Vario EL cube interfaced with an Elementar VisION IRMS, Elementar Analysensysteme GmbH, Langenselbold, Germany) at the University of California, Davis, Stable Isotope Facility. Analytical methods for the Cube system are described on the facility's website.

We analyzed total phosphorus concentration in fish tissue as concentration of ^31^P with the Thermo 2 inductively coupled plasma mass spectrometry system (ICP‐MS) in the Geochemistry Lab at the National High Magnetic Field Laboratory in Tallahassee, Florida. We prepared samples by dissolving them in nitric acid, following protocols described in Cooper et al. (2005) and Wilschefski and Baxter (2019), using 1 ppm phosphorus and 2 ppb indium as internal standards.

We did not have sufficient dried material from the periphyton samples to analyze their phosphorus content as well as the carbon and nitrogen levels. We gave priority to the carbon and nitrogen analyses in order to have baselines for estimating fish trophic position.

### 2.4 Calculation of Baseline δ^15^N Values and *H. formosa* Trophic Position

We estimated the trophic position of individual *H. formosa* by correcting for the variation in baseline *δ* ^15^N values of the periphyton separately for each site (trophic level 1; Vander Zanden and Rasmussen (1999)). We used the equation

$$\text{Trophic Position}=\left[\left(\delta^{15}N-\delta^{15}N_{\text{baseline}}\right)/3.4\right]+1$$

where 3.4% is the mean enrichment of *δ* ^15^N between trophic levels as determined in previous studies of fish that have the same range of longevity and tissue turnover rates as fish species in the sites we sampled (Aresco et al. 2015).

#### 2.4.1 Replication Statement

**Table:** 

Scale of inference	Scale at which the factor of interest is applied	Number of replicates at the appropriate scale
Population	Population	4 freshwater springs, 4 shallow lakes

#### 2.4.2 Statistical Analyses

We restricted our analyses to females for two reasons. First, this minimizes potential complications induced by sexual dimorphism in diet or growth patterns. Second, the greater range of body sizes in females offered a better opportunity to investigate whether elemental composition or trophic position varied with body size.

We tested the repeatability of water chemistry variation among the eight locations by calculating the pairwise correlations of values for each location between each pair of years for which we had data. We used one‐tailed tests for the significance of these correlations, inasmuch as only positive correlations indicate repeatable variation among locations. We used principal component analyses (PCA) to summarize water chemistry variation data by year, site, and water source, using the ‘ggord’ package (1.1.8) of R (R_Core_Team 2020).

Other North Florida open canopy lakes and springs habitats were included alongside the 8 focal sites to demonstrate our choice of sites spanned a wide range of water chemistry parameters not from the extremes of water quality variation.

We examined relationships of *δ* ^13^C, trophic position, and elemental phenotypes with body size via regressions of those variables on standard length. In 7 of the 8 populations, there was no relationship between an individual's standard length and its value of *δ* ^13^C (Figure S1A and Table S2). Likewise, there was considerable heterogeneity among the populations in their relationships of trophic position to standard length (Table S3). However, for this variable, slopes were small and there was no population in which individuals of different body lengths occupied different trophic positions (i.e., primary consumer, secondary consumer, etc.: Figure S1B). For these reasons, as well as the fact that the distributions of body length were similar in these populations, we performed our statistical analyses on population averages without adjusting for standard length.

We used analyses of variance (ANOVA) and Bonferroni pairwise comparisons to determine differences in elemental composition, stoichiometric ratios, and trophic position among populations of *H. formosa*. We examined residuals to confirm approximate normality, no dependence of variance on mean, and no directional trend in residual values. In addition, we applied Mantel analyses within the Vegan (2.6–2) package in R version (4.2.1) to test for significant association, through permutations, between matrices of Euclidean distances between pairs of populations in water quality, periphyton, and fish elemental phenotypes. We used 999 permutations in each Mantel analysis.

We did not adjust the raw *δ* ^13^C values for the lipid content of fish tissue. Mathematical corrections that use the C:N ratios as proxies for lipid content are in order when those ratios vary widely and when C:N values are above 4–5 (indicating lipid‐rich tissue) (Logan et al. 2008). In our case, average C:N ratios varied among populations and years between 4.18 and 5.45 (Table 2) and, in 2013, when we had large sample sizes, were not significantly associated with average values of *δ* ^13^C (*r* = −0.27, *p* > 0.25). Variation within populations in C:N ratios was also low, with coefficients of variation ranging between 0.04 and 0.09.

**TABLE 2:** Average values of elemental composition of fish and algal samples with standard errors.

Variable	Harpers Eyelet	Little Lake Jackson	Moore Lake	McBrides Slough	Natural Bridge	Shepherds Spring	Trout Pond	Wacissa River
Fish % C 2012	51.1 ± 8.5 (8)	—	41.2 ± 2.2 (9)	46.7 ± 6.0 (10)	—	45.2 ± 3.3 (8)	40.5 ± 1.8 (9)	44.4 ± 2.7 (9)
Fish % C 2013	38.4 ± 0.2 (61)	36.9 ± 0.3 (31)	39.3 ± 0.2 (36)	37.9 ± 0.3 (47)	39.2 ± 0.4 (11)	40.6 ± 0.2 (76)	39.9 ± 0.2 (31)	40.1 ± 0.2 (54)
Fish % N 2012	10.8 ± 1.6 (8)	—	9.7 ± 0.5 (9)	11.4 ± 1.7 (10)	—	10.4 ± 0.7 (8)	9.4 ± 0.3 (9)	10.0 ± 0.6 (9)
Fish % N 2013	10.7 ± 0.03 (61)	9.4 ± 0.15 (31)	10.5 ± 0.09 (36)	10.2 ± 0.04 (47)	10.1 ± 0.04 (11)	10.1 ± 0.04 (76)	11.0 ± 0.05 (31)	10.6 ± 0.04 (54)
Fish % P 2012	2.3 ± 0.1 (8)	—	2.3 ± 0.2 (9)	2.7 ± 0.3 (10)	—	2.9 ± 0.1 (8)	2.8 ± 0.2 (9)	2.5 ± 0.1 (9)
Fish % P 2013	2.9 ± 0.06 (61)	3.5 ± 0.08 (31)	2.7 ± 0.06 (36)	3.5 ± 0.30 (47)	2.6 ± 0.22 (11)	2.8 ± 0.05 (76)	2.6 ± 0.06 (31)	2.4 ± 0.06 (54)
Periphyton % C 2013	25.5 ± 1.86 (2)	19.4 ± 0.03 (2)	39.3 ± 1.50 (2)	22.5 ± 1.44 (2)	27.4 ± 0.09 (2)	29.3 ± 0.72 (2)	33.7 ± 0.21 (2)	29.0 ± 0.96 (2)
Periphyton % N 2013	1.6 ± 0.12 (2)	1.2 ± 0.01 (2)	2.8 ± 0.19 (2)	2.2 ± 0.27 (2)	2.5 ± 0.01 (2)	4.5 ± 0.09 (2)	2.1 ± 0.03 (2)	2.9 ± 0.12 (2)
Fish C:N 2012	5.45 ± 0.13 (8)	—	4.80 ± 0.09 (9)	5.19 ± 0.07 (10)	—	5.05 ± 0.16 (8)	4.78 ± 0.06 (9)	5.04 ± 0.09 (9)
Fish C:N 2013	4.18 ± 0.01 (61)	4.59 ± 0.06 (31)	4.38 ± 0.03 (36)	4.35 ± 0.03 (47)	4.52 ± 0.07 (11)	4.68 ± 0.03 (76)	4.24 ± 0.02 (31)	4.41 ± 0.02 (54)
Fish C:P 2012	59.33 ± 10.68 (8)	—	46.92 ± 6.63 (9)	38.94 ± 2.70 (10)	—	39.77 ± 5.04 (8)	45.96 ± 5.59 (9)	40.12 ± 2.20 (9)
Fish C:P 2013	39.40 ± 4.56 (61)	27.92 ± 0.82 (31)	38.61 ± 1.28 (36)	31.14 ± 1.27 (47)	47.39 ± 11.28 (11)	38.82 ± 0.77 (76)	39.95 ± 1.11 (31)	49.36 ± 6.79 (54)
Fish N:P 2012	10.71 ± 1.76 (8)	—	9.79 ± 1.45 (9)	7.47 ± 0.44 (10)	—	7.93 ± 1.10 (8)	9.61 ± 1.18 (9)	7.96 ± 0.42 (9)
Fish N:P 2013	9.37 ± 1.04 (61)	6.13 ± 0.22 (31)	8.80 ± 0.27 (36)	7.11 ± 0.26 (47)	10.42 ± 2.41 (11)	8.26 ± 0.14 (76)	9.39 ± 0.24 (31)	11.15 ± 1.51 (54)

Within the range of C:N values we obtained, the correction advocated by Skinner et al. (2016) for fish muscle tissue, which converts C:N ratios to percentage lipid using the formula of Post et al. (2007) and uses those values in the bias adjustment derived in Kiljunen et al. (2006), is linear and moves values of *δ* ^13^C by about 2 ppt (parts per thousand). A small linear adjustment merely shifts all population averages toward more enriched values by the same amount. In this light, we retained the raw values in order to avoid increasing the overall variance in *δ* ^13^C values via the propagation of variance through linear transformations (Travis 1982).

## 3 Results

### 3.1 Water Quality

Concentrations of chlorophyll a and total nitrogen were consistently above our detection limits while concentrations of nitrate were often, but not always, above our detection limits (0.012 mg/L). Those variables and pH varied widely among locations and, in some cases, across years (Table 1). Variation among locations in the concentration of nitrate and pH was repeatable across all 4 years; the six pairwise correlations between years for nitrate ranged from 0.72 to 0.98 (all *p* < 0.005) and, for pH, ranged from 0.93 to 0.98 (all *p* < 0.005). The variation in concentration of chlorophyll a was repeatable between 2010 and 2011 (*r* = 0.70, *p* < 0.01) and between 2010 and 2012 (*r* = 0.60, *p* < 0.01) but not between other pairs of years. There was a similar pattern of variation among locations in the concentration of total nitrogen; the variation was repeatable between 2010 and 2011 (*r* = 0.60, *p* < 0.01) and between 2011 and 2013 (*r* = 0.71, *p* < 0.01) but not between the other pairs of years.

**FIGURE 1:**
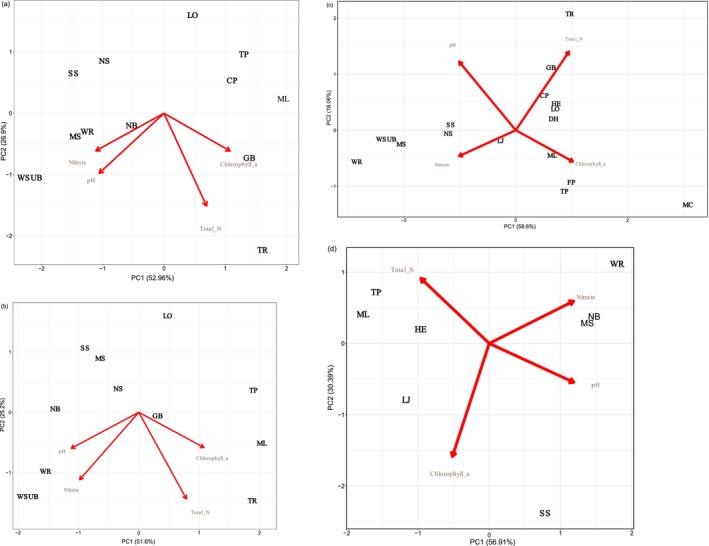
Principal component analysis of water chemistry parameters for four years: 2010 (a), 2011 (b), 2012 (c), and 2013 (d). Other North Florida open canopy lakes and springs habitats are shown alongside the 8 focal sites to demonstrate our choice of sites spanned a wide range of water chemistry parameters not from the extremes of water quality variation.

Principal components analyses of these four variables produced a consistent pattern in all 4 years (Figure 1). The first axis described a gradient from lakes with lower values of pH, higher concentrations of chlorophyll a, and lower nitrate concentrations to springs with higher values of pH, lower chlorophyll a concentrations, and higher nitrate concentrations. The second axis usually described a gradient in total nitrogen concentration, which did not separate lake and spring locations. In 2013, the concentration of chlorophyll a contributed to this axis. The individual locations retained their positions on the first axis, relative to one another, across the 4 years, although they did not retain their relative positions on the second axis with the same consistency.

### 3.2 Stoichiometry

In neither year did the elemental composition of the fish vary substantially in magnitude among populations (Table 2). Among populations, the average percentages of nitrogen, by mass, varied between about 9% and 11% in each year. The average percentages of phosphorus varied between about 2.3% and 3.5% overall. The average percentages of carbon varied more among locations in 2012 (40%–51%) than in 2013 (37%–40%). The average values of percentage carbon were lower in 2013 than in 2012 and, accordingly, the average percentages of nitrogen and phosphorus were slightly higher in 2013. In 2013, when sample sizes were large, there were no significant relationships with body length for %N (*r* = −0.00004, *p* = 0.99), %C (*r* = 0.10, *p* = 0.04), or %P (*r* = 0.02, *p* = 0.67).

The low level of variation in elemental composition translated into low levels of variation in the stoichiometric ratios among populations (Table 2). There were no strongly significant relationships between body length and any of the three stoichiometric variables: C:N (*r* = 0.14, *p* = 0.05); C:P (*r* = −0.03, *p* = 0.58); and N:P (*r* = −0.08, *p* = 0.09).

In 2012, variation within each population in C:N ratios was sufficiently small that the average C:N ratios differed significantly among populations (*F*_5,46_ = 5.97, *p* < 0.001). The Bonferroni multiple comparison indicated that there were three groups: two lakes, Trout Pond and Moore Lake, with the lowest ratios; three springs, Wacissa River, Shepherd's Spring, and McBride's Slough, with intermediate ratios; and one lake, Harper's Eyelet, which had the largest ratio. There were no significant differences in average C:P (*F*_5,46_ = 1.27, *p* = 0.25) or N:P ratios (*F*_5,46_ = 1.06, *p* = 0.40).

The much larger sample sizes of fish in 2013, compared with 2012, contributed to more robust conclusions (Halvorson and Small 2016), making some populations stand apart statistically from others in some ratios, although the overall variation among population averages remained small. There was a significant difference among populations in average C:N ratios (*F*_7339_ = 37.09, *p* < 0.001); the Bonferroni multiple comparison indicated that the C:N ratios at Shepherd's Spring and Little Lake Jackson were larger than the others, with the ratio at Harper's Eyelet being below the others. This pattern of differences among populations was not consistent with the pattern in 2012; Harper's Eyelet had the largest ratio in 2012 but the smallest in 2013, whereas Shepherd's Spring had an intermediate ratio in 2012 but one of the highest in 2013. Unlike in 2012, there was a significant difference in average C:P ratios in 2013 (*F*_7339_ = 2.77, *p* = 0.008); in this case, multiple comparison tests indicated only that Wacissa River differed from Little Lake Jackson and McBride's Slough. A similar pattern emerged from the analysis of N:P ratios, although the overall test was barely significant (*F*_7339_ = 3.09, *p* = 0.04).

**FIGURE 2:**
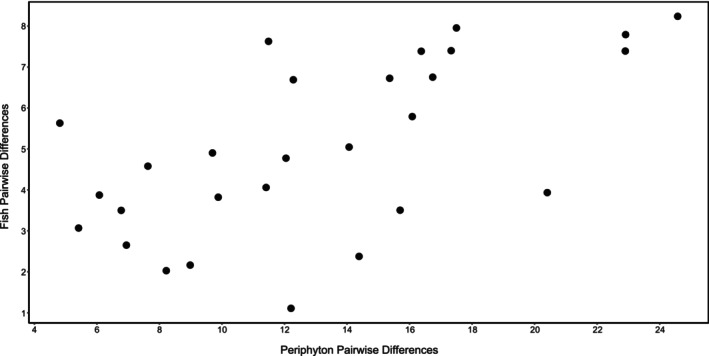
Relationship between pairwise differences in elemental composition of periphyton and pairwise differences in elemental composition of *Heterandria formosa* across eight populations in north Florida.

The Mantel analyses revealed little evidence of associations between elemental composition of fish and water quality variables but did reveal an association between the elemental compositions of fish and periphyton. The differences between pairs of populations in elemental compositions of fish were not correlated with differences in the water quality variables in either 2012 (*r* = 0.1468, *p* = 0.171) or 2013 (*r* = 0.1414, *p* = 0.179). The differences between pairs of populations in the percentages of carbon and nitrogen in fish were moderately correlated with the pairwise differences in those variables in the periphyton samples in 2013 (*r* = 0.61, *p* < 0.001, Figure 2). In 2012, the same correlation was weaker and not statistically significant (*r* = 0.32, *p* = 0.08).

### 3.3 Periphyton Isotope Samples

**FIGURE 3:**
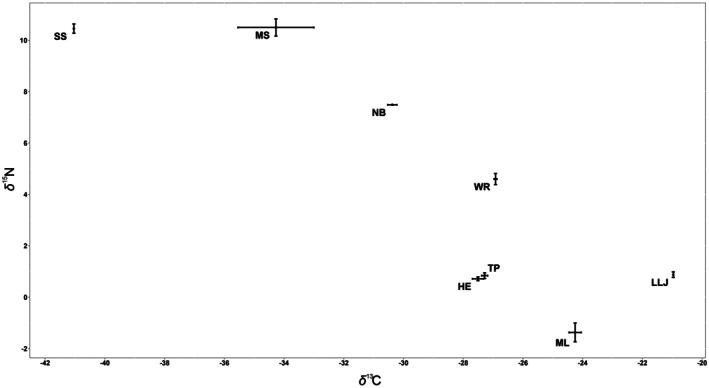
Average values of *δ* ^13^C and *δ* ^15^N for periphyton samples with standard errors (when multiple samples were obtained) for eight locations in north Florida.

Average values of *δ* ^13^C ranged widely among locations, with a tendency for periphyton samples from springs to be less enriched in *δ* ^13^C and those from lakes to be more enriched (Figure 3). The average values for the shallow springs, Shepherd's Spring and McBride's Slough, were much lower than the values for the other locations. There were significant differences among locations (*F*_7,8_ = 179.36, *p* < 0.001), with a Bonferroni multiple comparison indicating that the average values in Shepherd's Spring and McBride's Slough were distinct from all the other locations.

The average values of *δ* ^15^N also varied widely among locations, with periphyton from springs displaying more enriched *δ* ^15^N than those from lakes (Figure 3). Periphyton from locations with less enriched *δ* ^15^N also had more enriched *δ* ^13^C, again reflecting the broad distinction between springs and lakes (correlation among average values of each variable *r* = 0.859, *p* < 0.01). The variation in average values of *δ* ^15^N was significantly different among locations (*F*_7,8_ = 497.39, *p* < 0.001), with the Bonferroni multiple comparison indicating that most locations were distinct from one another; Shepherd's Spring and McBride's Slough were indistinguishable from each other, as were the trio of Little Lake Jackson, Trout Pond, and Harper's Eyelet. Moore Lake stood apart from the others in having a negative value of *δ* ^15^N; this suggests the presence of cyanobacteria engaged in fixation of atmospheric N_2_ (Yoneyama et al. 1991; Helmer et al. 2024).

### 3.4 Fish Isotope Samples

**FIGURE 4:**
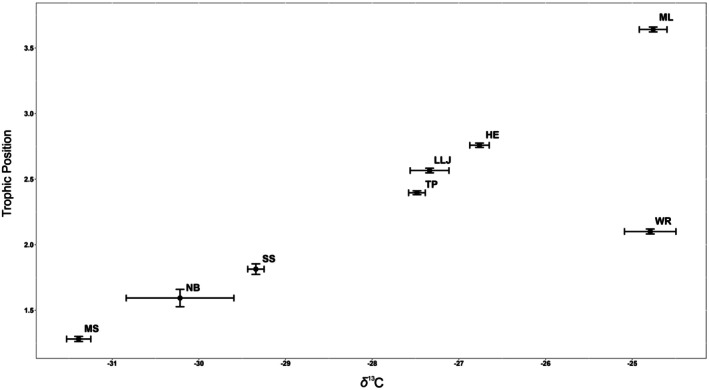
Average values of *δ* ^13^C and trophic position with standard errors for fish from eight populations of *Heterandria formosa* in north Florida.

**FIGURE 5:**
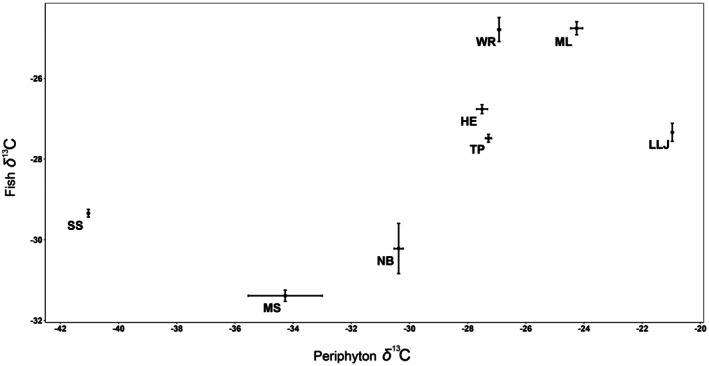
Average values of *δ* ^13^C with standard errors for periphyton and *Heterandria formosa* from eight populations in north Florida.

There was substantial variation among populations in the 2013 samples in their average values of *δ* ^13^C (Figure 4). The differences in these averages were statistically significant (*F*_7339_ = 161.90, *p* < 0.001). The Bonferroni multiple comparison revealed a gradation of population averages. The trio of small springs, McBride's Slough, Natural Bridge, and Shepherd's Spring, displayed low levels of enrichment and were statistically distinguishable from all other populations. McBride's Slough was indistinguishable from Natural Bridge but distinguishable from Shepherd's Spring, which could not be distinguished from Natural Bridge. The trio of lentic systems, Harper's Eyelet, Little Lake Jackson, and Trout Pond, displayed moderate levels of enrichment. These populations were statistically indistinguishable from one another but, as a group, were distinguishable from all other populations. The two largest sites, Moore Lake and Wacissa River, were indistinguishable from each other but distinguishable from all others. The average values of *δ* ^13^C in the fish populations were highly correlated with the average values of *δ* ^13^C in the periphyton samples at those same locations (*r* = 0.64, *p* < 0.025, Figure 5), indicating that periphyton are the primary source of carbon in the diets of the fish.

Populations also differed substantially in their average values of *δ* ^15^N (Figure S2). At the extremes, the average value for Shepherd's Spring, which had the highest average value of *δ* ^15^N, was over twice that for Trout Pond, which had the lowest value among the eight populations. These averages were strongly correlated with the average *δ* ^15^N values for the periphyton collected at each location (*r* = 0.91, *p* = 0.002). There was a strong statistical difference among the average *δ* ^15^N values for the fish in these populations (*F*_7339_ = 833.12, *p* < 0.001). The Bonferroni multiple comparison could not distinguish Trout Pond from Little Lake Jackson or Harper's Eyelet from Little Lake Jackson; all other pairwise differences among these populations were significant.

The average trophic positions of fish in all populations except Moore Lake varied between 1.5 and 3, with Moore Lake fish displaying an average above 3.5 (Figure 4). The trophic position of fish in Moore Lake is likely to be artificially inflated because of the negative value of *δ* ^15^N estimated in the periphyton samples (Figure 3). The average values of trophic position in these 8 populations were significantly different from one another (*F*_7339_ = 549.65, *p* < 0.001). The Bonferroni multiple comparison indicated that each population was distinct from every other population.

Aside from Moore Lake, the average values displayed a clear pattern, with fish in the small springs having the lowest trophic position, largely (McBride's Slough) but not entirely (Natural Bridge, Shepherd's Spring) as primary consumers, the fish in the remaining lakes (Trout Pond, Little Lake Jackson, Harper's Eyelet) having a higher trophic position as secondary consumers, and with fish in the large spring (Wacissa River) having a trophic position intermediate between these two groups. There was a positive association among the populations between their average values of trophic position and their average values of *δ* ^13^C (*r* = 0.81), which would be stronger in the absence of the Wacissa River population (Figure 4). This association reflects the inverse relationship found among locations in the periphyton samples, in which samples more enriched in *δ* ^13^C were less enriched in *δ* ^15^N which, all else equal, would force the estimation of trophic position upward.

There was a tendency for populations with higher average trophic positions to have lower average C:N ratios (*r* = −0.29), although this correlation was not significant. There was no evidence that average trophic position in a population was correlated either with its average N:P ratio (*r* = −0.05) or average C:P ratio (*r* = −0.16).

## 4 Discussion

While there was little variation in elemental composition among these eight populations of *H. formosa*, that variation tracked the variation observed in the periphyton samples from their respective locations. There was wider variation among the fish populations in their levels of *δ* ^13^C; which also tracked variation in the *δ* ^13^C levels in the periphyton samples. These results are nicely aligned with the conclusion of Aresco et al. (2015) that periphyton form the resource base for the aquatic food web in which *H. formosa* is embedded.

There was similar variation among populations in average trophic position, from populations that can be considered primary consumers (McBride's Slough, Natural Bridge, and Shepherd's Spring) to ones that can be considered secondary consumers (all others). The negative values of *δ* ^15^N at Moore Lake probably inflated its average trophic position artificially. However, it seems clear that even if this is so, the fish at Moore Lake ought to be considered secondary consumers. In light of the variation in trophic position, it is noteworthy that there was no associated variation in elemental composition. The lack of association contrasts with interspecific patterns (Vanni et al. 2002; McIntyre and Flecker 2010), perhaps because species included in these meta‐analyses vary more widely in trophic position than do populations of the same species.

In a larger perspective, the elemental traits we estimated in periphyton are similar to the results for epilithic algae reported from Trinidadian streams and north Florida lakes, in which similar analyses have been completed (Kohler et al. 2012). The elemental composition of *H. formosa* is comparable to that in another poeciliid fish, the Trinidadian guppy ( *Poecilia reticulata* ) (El‐Sabaawi, Zandona, et al. 2012). Our data show that *H. formosa* had a comparable percentage of carbon to *P. reticulata* but slightly higher nitrogen percentages and slightly lower phosphorus percentages. Accordingly, the C:N ratios for *H. formosa* were slightly lower than those of *P. reticulata* , while the C:P and N:P ratios were slightly higher.

While water chemistry varied markedly among our locations, our data are comparable to previous results reported for water chemistry in north Florida (Aresco 2009). Likewise, that variation was not associated with variation in the elemental composition of the fish. The variation in water chemistry was consistent with well‐known abiotic differences between springs and lakes (Leips and Travis 1999). There was a noticeable, but non‐significant, association between pairwise population differences in water chemistry and the carbon and nitrogen composition of periphyton (*r* = 0.32, *p* = 0.08). In north Florida, the species of diatoms and soft algae in the periphyton change markedly along gradients of pH (Whitmore 1989; Sweets et al. 1990; Mattson et al. 1995; Stevenson et al. 2004; Fore 2010). This suggests that the weak association we found between water chemistry and organismal stoichiometry of periphyton could result from changes in species composition of the periphyton along the pH gradient between springs and lakes.

There are two hypotheses for the association between the elemental composition of fish and periphyton, which are not mutually exclusive. First, the fish may not have a tightly regulated homeostasis, which would create the association in a bottom‐up fashion (Schade et al. 2003; Dickman et al. 2008; Zandonà et al. 2024). Second, the fish could be influencing nutrient recycling rates and creating a feedback to the periphyton via excretion and decomposition, creating the association via a top‐down effect (Evans‐White and Lamberti 2006; Taylor et al. 2012). The relative importance of each factor can only be ascertained by mesocosm experiments that manipulate the composition of the basal resources (Halvorson and Small 2016; López‐Sepulcre et al. 2024) and the amount of excretion into the ecosystem (Bassar et al. 2012).

While we found significant population variation in elemental traits, that variation was different from prior work on intraspecific variation in two respects. First, populations of *H. formosa* displayed less variation than has been described in comparably sized surveys of other small‐bodied fish (El‐Sabaawi, Zandona, et al. 2012; El‐Sabaawi, Bassar, et al. 2015; Durston and El‐Sabaawi 2017). This may reflect less divergence in the composition of the basal resources exploited by *H. formosa* than the divergence in resource composition in other systems. This is a hypothesis that remains to be tested. Second, whereas several other surveys found that N:P ratios were most variable among populations (Vrede et al. 2011; Tuckett et al. 2016; Durston and El‐Sabaawi 2017), we found that the C:N ratios were most variable. This may reflect variation among *H. formosa* populations in life history, especially reproductive allocation, which can influence C:N ratios (El‐Sabaawi, Kohler, et al. 2012).

The populations in our survey that experience the higher predation risks, as described in previous work (MacRae and Travis 2014), here Little Lake Jackson, Moore Lake, and Trout Pond, had higher average values of trophic position. While we do not have quantitative data on predator abundance at Harper's Eyelet, the predator fauna is similar to that of Moore Lake. Lake populations have higher predator densities, on average, than do spring populations (Leips and Travis 1999; MacRae and Travis 2014). This relationship between higher predation intensity and feeding higher in the trophic web is similar to the distinction between high‐ and low‐predation populations of guppies, *P. reticulata* (Zandona et al. 2011). In both guppies and *H. formosa*, lower predation pressure is associated with higher densities and greater intraspecific competition for food. Higher predation relaxes the influence of competition on those that escape predators, allowing them to feed higher in the trophic web on higher‐quality food, in both cases aquatic invertebrates, instead of periphyton and detritus. However, we found no difference in elemental composition with variation in trophic position, whereas guppies feeding higher in the trophic web had higher percentages of nitrogen (Zandonà et al. 2024). The difference may stem from the fact that when guppies are feeding higher, they are consuming aquatic insects and small macroinvertebrates, whereas when *H. formosa* do so, they are largely consuming small cladocerans (Reimer 1970; Schaefer et al. 1994).

Our results illuminate several ecological issues. First, they reinforce work showing that alleviation of predation and elevation of density can cause omnivorous fish to feed higher in the trophic web. Second, they show how the elemental composition of a fish low in the trophic web tracks the elemental composition of its resource base. Third, they show that the variation in trophic position among populations is not associated with variation in elemental composition. Together, the results suggest the primacy of the composition of the resource base over that of trophic ecology in governing population variation in elemental composition.

## Author Contributions

**Benjamin D. Pluer:** data curation (supporting), formal analysis (lead), investigation (equal), software (equal), validation (equal), visualization (lead), writing – original draft (supporting), writing – review and editing (equal). **Pamela S. D. MacRae:** conceptualization (equal), data curation (equal). **Joseph Travis:** conceptualization (lead), data curation (supporting), formal analysis (supporting), funding acquisition (lead), investigation (equal), methodology (equal), project administration (lead), resources (lead), software (equal), supervision (lead), validation (equal), visualization (supporting), writing – original draft (lead), writing – review and editing (equal).

## Conflicts of Interest

The authors declare no conflicts of interest.

## Supporting information


**Data S1:** Supporting Information.

## Data Availability

Data available from Dryad Digital Repository: https://doi.org/10.5061/dryad.3n5tb2rt5.
