# Supplementary material for: Intraspecific Variation in Elemental Composition of the Least Killifish Tracks Spatial Variation in Periphyton Composition
Source: Ecol Evol. 2025 Oct 13;15(10):e72326. doi: 10.1002/ece3.72326 (PMC12518787; doi:10.1002/ece3.72326)
Supplement: Supplementary file 1 — Data S1: Supporting Information. [file ECE3-15-e72326-s001.docx]

**Supplemental Tables**

| **Location** | **Abbreviation** | **Habitat** | **Latitude** | **Longitude** |
| --- | --- | --- | --- | --- |
| Harper’s Eyelet | HE | Open lake | 29.004217 | -84.409317 |
| Little Lake Jackson | LLJ | Open lake | 30.525782 | -84.358788 |
| Moore Lake | ML | Open lake | 30.392275 | -84.407272 |
| McBride’s Slough | MS | Spring | 30.239326 | -84.269539 |
| Natural Bridge | NB | Spring | 30.283352 | -84.150986 |
| Newport Spring | NS | Spring | 30.206261 | -84.178909 |
| Shepherd’s Spring | SS | Spring | 30.123451 | -84.280586 |
| Trout Pond | TP | Open lake | 30.334175 | -84.385086 |
| Wacissa River | WR | Spring | 30.339916 | -83.991203 |

**Supplemental Table 1.** Locations for sampling of water quality with abbreviations used in other tables and text, habitat, latitude, and longitude. Location names given in italics are those chosen for further stoichiometric and isotope analyses.

| Location | Number of individuals | Slope + Standard Error |
| --- | --- | --- |
| Harper’s Eyelet | 60 | 0.086 + 0.061 |
| Little Lake Jackson | 30 | 0.273 + 0.082* |
| Moore Lake | 35 | -0.048 + 0.047 |
| McBride’s Slough | 46 | -0.058 + 0.064 |
| Natural Bridge | 10 | -0.367 + 0.323 |
| Shepherd’s Spring | 75 | -0.048 + 0.049 |
| Trout Pond | 30 | 0.006 + 0.040 |
| Wacissa River | 53 | 0.207 + 0.109 |

**Supplemental Table 2.** Slopes of regressions of values of *δ*^13^C on standard length for individuals in each population. Asterisk denotes statistical significance at or below the 0.05 critical value.

| Location | Number of individuals | Slope + Standard Error |
| --- | --- | --- |
| Harper’s Eyelet | 60 | 0.009 + 0.009 |
| Little Lake Jackson | 30 | -0.005 + 0.007 |
| Moore Lake | 35 | -0.021 + 0.004* |
| McBride’s Slough | 46 | -0.026 + 0.008* |
| Natural Bridge | 10 | -0.032 + 0.035 |
| Shepherd’s Spring | 75 | -0.074 + 0.019* |
| Trout Pond | 30 | 0.015 + 0.005* |
| Wacissa River | 53 | -0.013 + 0.007 |

**Supplemental Table 3.** Slopes of regressions of values of trophic position on standard length for individuals in each population. Asterisk denotes statistical significance at or below the 0.05 critical value.

**Supplemental Figures**


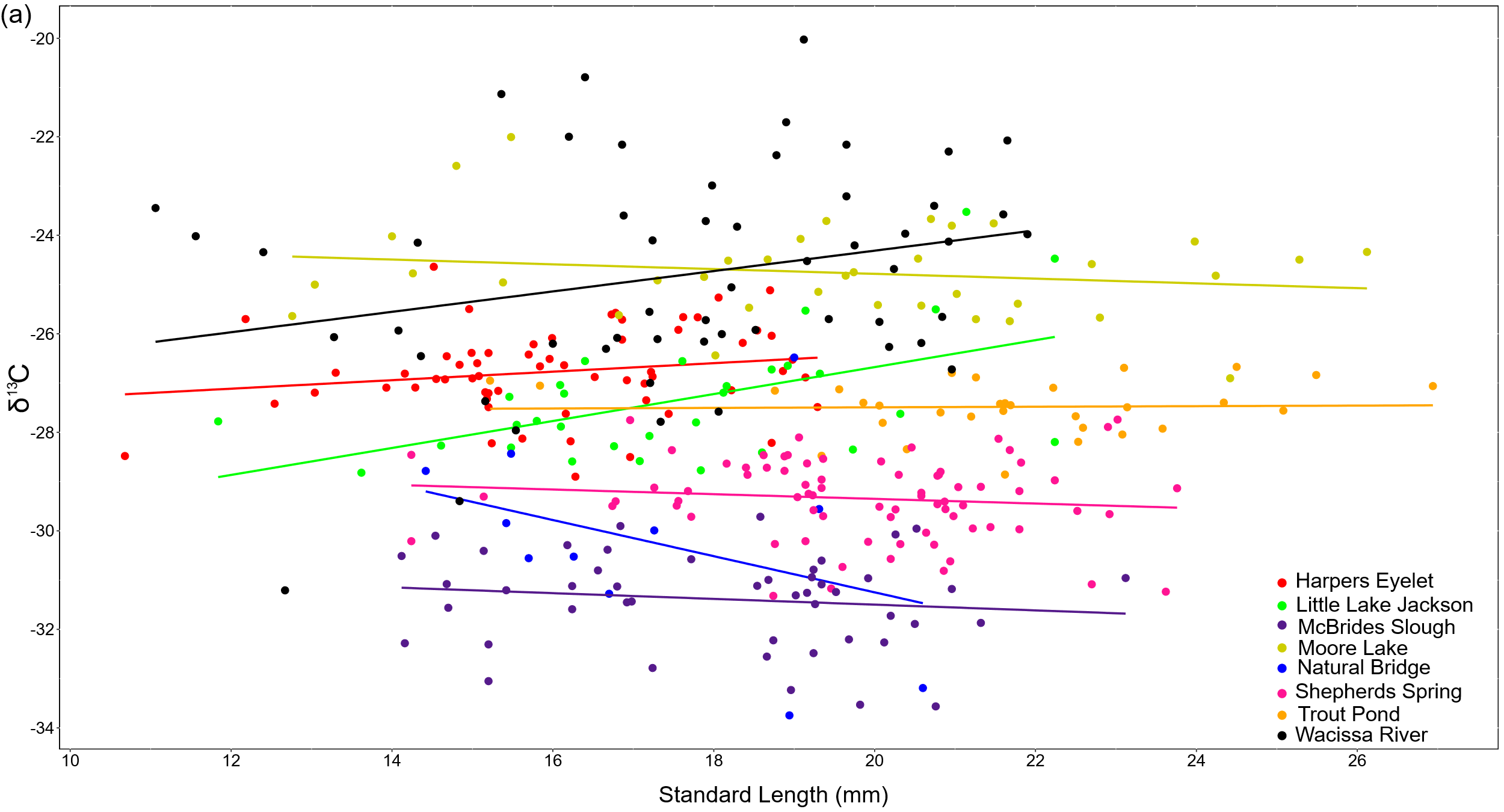


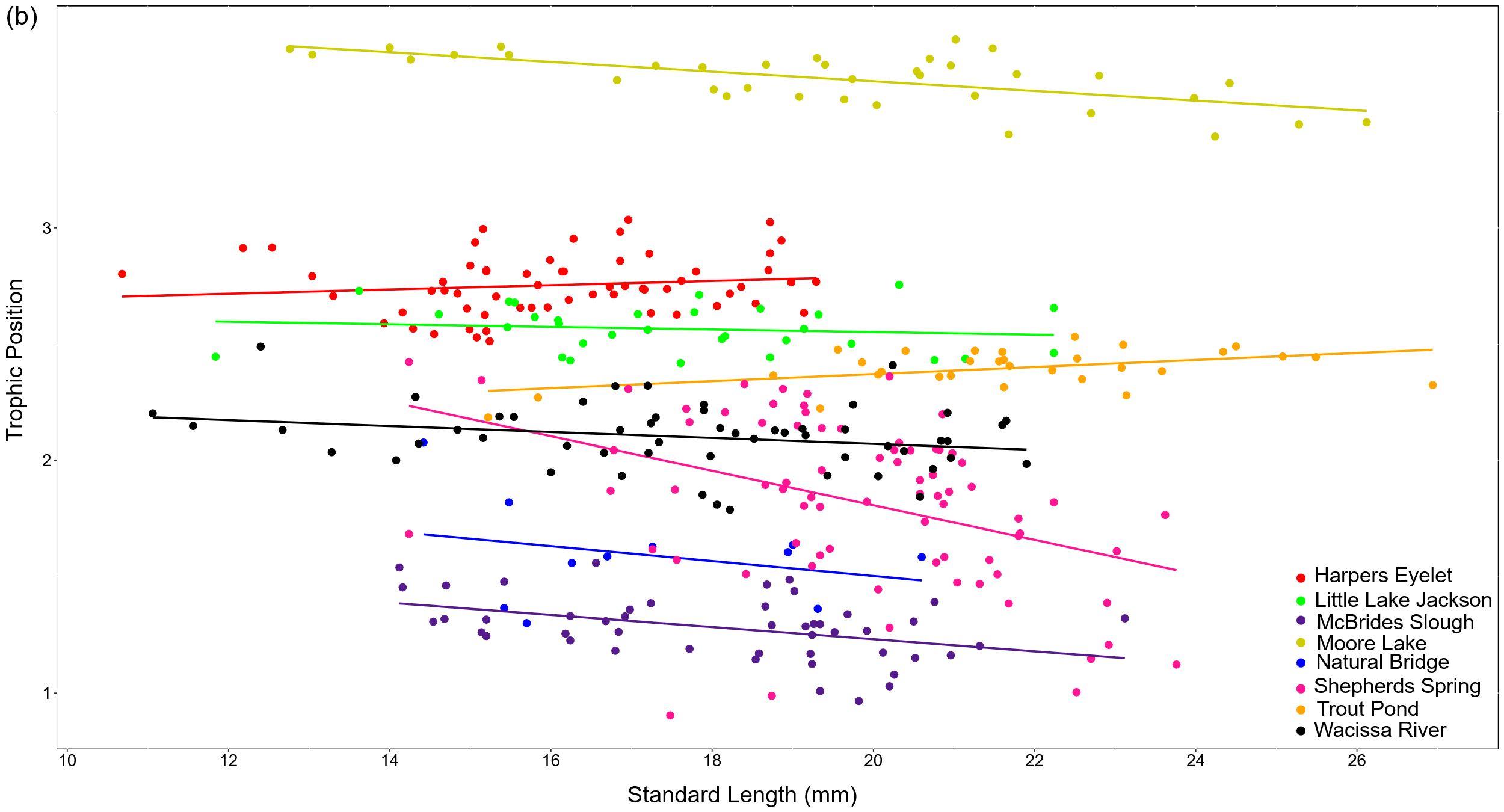


Supplemental Figure 1. Relationships between *δ*^13^C and standard length (a) and trophic position and standard length (b) for individuals from eight populations of *Heterandria formosa* in north Florida.

Supplemental Figure 2. Average values of *δ*^15^N with standard errors for *Heterandria formosa* from eight populations in north Florida.
